# Prevalence of Cardiovascular Risk Factors in the Middle-Aged and Elderly Population of a Nigerian Rural Community

**DOI:** 10.1155/2011/308687

**Published:** 2011-04-05

**Authors:** E. C. Ejim, C. I. Okafor, A. Emehel, A. U. Mbah, U. Onyia, T. Egwuonwu, J. Akabueze, B. J. Onwubere

**Affiliations:** ^1^Department of Medicine, University of Nigeria Teaching Hospital, Enugu PMB 01129, Nigeria; ^2^Department of Community Medicine, University of Nigeria, Enugu, Nigeria; ^3^82 Division Nigerian Army Hospital, Enugu, Nigeria

## Abstract

Cardiovascular diseases (CVDs) causes of worldwide preventable morbidity and mortality. CVDs are a leading cause of mortality and morbidity in developing countries, and rates are expected to rise over the next few decades. The prevalence of CVD risk factors is dramatically increasing in low-and middle-income African countries, particularly in urban areas. We carried out a cross-sectional population-based survey in Imezi-Owa, a rural community in South East Nigeria to estimate the prevalence of major cardiovascular risk factors in both men and women aged 40–70 years. A total of 858 individuals made up of 247 (28.8%) males and 611 (71.2%) females were recruited. The mean age of the subjects was 59.8 ± 9.9 years. The prevalence of the different cardiovascular risk factors among the 858 subjects was as follows: hypertension 398 (46.4%) subjects, generalized obesity as determined by BMI 257 (30%) subjects, abdominal obesity 266 (31%) subjects, dysglycaemia 38 (4.4%) subjects and hypercholesterolaemia 32 (3.7%) subjects. Prevalence of hypertension and dysglycaemia was higher in men while the others were higher in women. Only hypertension (*P* = .117) and hypercholesterolaemia (*P* = .183) did not reveal any significant association with gender. Prevalence of CVD risk factors was highest in subjects aged 65 to 70 years.

## 1. Introduction

Cardiovascular diseases (CVDs) are important causes of worldwide preventable morbidity and mortality [1, 2]. CVDs have become a leading cause of mortality and morbidity in developing countries and rates are expected to rise further over the next few decades [3]. Relative to white subjects, Afro-Caribbean and people of African descent have high incidence of stroke and end stage renal failure whereas coronary heart disease is less common [4–6]. Once considered a problem only in high-income countries, the prevalence of CVD risk factors is dramatically increasing in low- and middle-income African countries, particularly in urban areas [7, 8]. Studies in Tanzania have reported high rates of hypertension in both urban and rural areas, particularly among the obese and elderly [9]. In Ghana, earlier studies revealed hypertension prevalence to be 4.5% among rural dwellers and 8% to 13% in the urban dwellers [10]. 

The estimated prevalence of diabetes mellitus in Sub-Saharan Africa is about 1% in rural areas and 5–7% in urban areas, and between 8% and 13% in countries like Uganda and South Africa [11–13]. Overweight and obesity are leading risk factors for a number of chronic diseases, including CVD, diabetes mellitus, and cancer. Obesity is a leading determinant of hypertension, dyslipidaemia, and diabetes mellitus [14]. 

In Ghana, earlier studies revealed a hypertension prevalence of 4.5% among rural dwellers while in Nigeria the prevalence of hypertension was found to be 10% in rural areas [10, 11].

A generally favourable lipid profile (low total and LDC cholesterol, and normal to high HDL cholesterol) and low homocysteine values have been reported among the general population in Africa [15, 16].

However, hyperlipidaemia is becoming increasingly common, and studies from Tanzania observed 25% prevalence of elevated serum total cholesterol (cholesterol > 5.2 mmol/L) [9] and 15% prevalence of elevated triglycerides (TG ≥ 1.7 mmol/L) among adults over 35 years of age, with women being affected more than men.

Nigeria is a multi ethnic nation organized into six geo-political zones inhabited by people with diverse cultures. Most of the rural communities are inhabited by children, men, and women who belong more to the low socioeconomic strata. Most of the men and women are elderly people who have retired from active service. Most of the active youths and middle-aged individuals reside in the urban and semi-urban cities characterized by better social amenities and job opportunities. Cardiovascular risk factors have been thought to be less common in these rural communities due to the traditional lifestyle the inhabitants are thought to adopt. 

We carried out a population-based survey in Imezi-Owa, South East Nigeria, to estimate the prevalence of major cardiovascular risk factors in both men and women aging 40–70 years.

## 2. Methodology, Study Design, and Population

This study was conducted in the two administrative wards of Imezi-Owa, a rural community in Enugu State, South East Nigeria. The community is inhabited by the Igbos, one of the major ethnic groups in Nigeria, and has a population of about 28,808 adults based on the national census figures of 2005. The community is predominantly dominated by children and their young/middle-aged mothers as well as the elderly men and women most of who have retired from active work. The young and middle-aged men live in the cities where they are more gainfully employed and visit the rural areas periodically. The few young and middle-aged men who reside in these rural areas are mostly subsistent farmers. A total of eight hundred households were randomly selected, and all the adults aging 40 to 70 years in these households were selected for the study. A total of 858 adults agreed to take part and reported for the study at the health centers. The response rate was 70.4%. The information sheet and consent form written in English were explained to the participants in the local language and all the consenting patients signed their names or applied a thumbprint. 

The research protocol was approved by the Ethics Committee of the University of Nigeria Teaching Hospital Ituku Ozalla, Enugu.

### 2.1. Data Collection

All measurements were conducted by two trained physicians and one nurse between 8:00 AM and 10:00 AM at designated health centres. Questionnaires were administered by the physicians and data obtained included age, gender, occupation, average monthly income, and history of cardiovascular disease.

Systemic blood pressure was measured using a standard mercury sphygmomanometer on the left arm after 5-minute rest using a cuff of appropriate size with the subject in the sitting position. The first and fifth phases of Korotkoff sounds were used for systolic (SBP) and diastolic blood pressures (DBP), respectively. Two independent measurements were obtained with a minimum interval of one minute [17].

Anthropometric measurements including height, weight, waist, and hip circumferences were obtained by the nurse. Height was measured without shoes to the nearest centimeter using a ruler attached to the wall, while weight was measured to the nearest 0.1 kg on an electronic scale with the subject wearing light outdoor clothing and no shoes. Waist circumference was measured at the highest point of the iliac crest with the subject in light clothing.

### 2.2. Blood Tests

The observation of a fasting state (which they had been earlier asked to observe) was ascertained from each participant, and blood samples were collected under aseptic procedures from a finger puncture. Dry chemistry tests were used to measure total cholesterol (TC) and fasting blood glucose level using the Accutrend GC System (Accutrend GC, Roche Diagnostics, Germany). Measuring ranges of the device for glucose were 1.1–33.3 mmol/L and for total cholesterol 3.88–7.76 mmol/L. The Accutrend GC System does not measure LDL cholesterol, HDL cholesterol, or VLDL cholesterol.

Precision for glucose was <3% and for cholesterol <5%. Accuracy of the Accutrend GC System for glucose test was ±5% compared to a hexokinase protein-free precipitate method and for cholesterol, and it was ±5% compared with cholesterol oxidase/P-aminophenazone (CHOP-PAP) method. Results for the glucose and cholesterol tests were obtained within 12 seconds and 180 seconds, respectively. Measuring principle of the device was reflectance photometry.

Sample volumes needed for the device were one drop of blood for cholesterol and one drop of blood for glucose applied directly from the fingertip.

The Accu-chek softclix prolancing device was used in the study.

### 2.3. Definition of Risk Factors

Hypertension was defined as systolic blood pressure ≥ 140 mmHg and/or diastolic blood pressure ≥ 90 mmHg [18] or being on pharmacological treatment for hypertension. The different grades of hypertension were defined as follows: mild (140/90 mmHg to 159/99 mmHg), moderate (160/100 to 179/109 mmHg), and severe hypertension (≥180/110 mmHg).

Participants were diagnosed to have diabetes mellitus (DM) if they had fasting blood glucose level (FBG) ≥ 7 mmol/L or reported a history of diabetes or use of glucose-lowering drugs [19]. Subjects with impaired fasting glycaemia (IFG) were defined by FBG ranging from 6.1 to 6.99 mmol/L. Subjects found to have DM or IFG were regarded to have abnormal glucose tolerance (dysglycaemia). Overweight and generalized obesity were defined as body mass index (BMI) ≥ 25 and 30 kg/m^2^, respectively. 

Abdominal obesity was defined as waist circumference of ≥102 cm in men and ≥88 cm in women [20]. High cholesterol was defined as greater than or equal to 6.2 mmol/L [21].

### 2.4. Statistical Analysis

Statistical analysis was done using the Personal Computer (PC) analytical software, Statistical Package for Social Sciences (SPSS Inc, Chicago, IL) version 17. Results were expressed as either mean values (standard deviation) or proportions, and comparison for statistical significance was by student's *t*-test for continuous variables or chi-square analysis for categorical variables. The level of significance level was set at *P* ≤ .05.

## 3. Results

A total of 858 individuals made up of 247 (28.8%) males and 611 (71.2%) females took part in the study. The characteristics of the population classified by gender are shown in Table 1. 

The prevalence of the different cardiovascular risk factors among the 858 subjects was as follows: hypertension 398 (46.4%) subjects, generalized obesity as determined by BMI 257 (30%) subjects, abdominal obesity 266 (31%) subjects, dysglycaemia 38 (4.4%) subjects, and hypercholesterolaemia 32 (3.7%) subjects. 

Among those identified as having hypertension, 197 (49.5%) subjects had mild hypertension while 20.4% had severe hypertension. The rest had moderate hypertension.

Prevalence of hypertension and dysglycaemia was higher in men while the others were higher in women. Only hypertension and hypercholesterolaemia did not reveal any significant association with gender. These are further shown in Table 2. 

Prevalence of hypertension increased with age whereas indices of obesity decreased with age. The associations with age were only statistically significant for hypertension (*χ* ^2^(5) = 14.88; *P* = .011), generalized (*χ* ^2^(5) = 35.58; *P* < .0001) and abdominal obesity (*χ* ^2^(5) = 18.03; *P* = .003). Abnormal glucose tolerance and hypercholesterolaemia did not show any significant trend (*P* > .05). 

**Figure 1:**
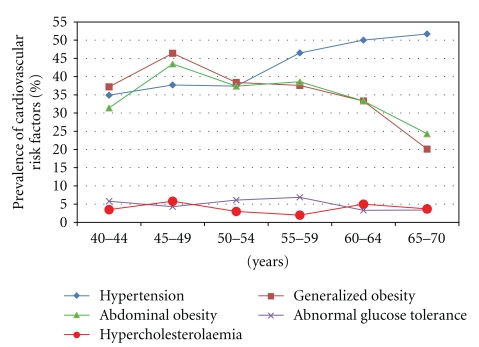
Trend of prevalence of cardiovascular risk factors within the age groups.

The trend of the prevalence of these cardiovascular risk factors is shown in Figure 1, while Table 3 shows the exact proportion of the risk factors within the age groups.

## 4. Discussion

This study was undertaken to evaluate the burden (prevalence) of major cardiovascular risk factors namely, hypertension, obesity (generalized and central), dysglycaemia, and hypercholesterolaemia among subjects aging 40 to 70 years who live in a rural community in Enugu State, South Eastern Nigeria. 

The gender bias in favour of females in this particular study may be explained by the nature of the community which is mainly inhabited by children and their young/middle-aged mothers as well as the elderly men and women most of who have retired from active work whereas the young/middle aged people are affected by rural-urban migration. Also the bias may be explained by one of the anthropological characteristics of the traditional Igbo society where health seeking is considered as a feminine behavior until an illness becomes severe. At such times, because of the importance attached to males as heads and sustainers of the family, they are quickly taken to hospitals. This is thought to explain the reversal of the above observation in some hospital-based studies where there may be male preponderance.

Among these subjects, hypertension and obesity recorded the highest prevalence whereas dysglycaemia and hypercholesterolaemia were still relatively low. As generally observed globally, though the prevalence of CVD risk factors has been shown to be on the increase both in Western and African countries, particularly in urban areas, the rural areas which have suffered from rural-urban migration are becoming affected too [7–9]. While most reports have emanated from hospital-based studies, community-based studies which truly describe the population are few. 

Compared to earlier reports from rural communities in Ghana and Nigeria [10, 11], rising trend in the prevalence of hypertension is thus well demonstrated. This may be masked if one goes by the mean values of the blood pressure variables which were all within normal limits (Table 1). The prevalence of hypertension in this study was 46.4% as against ≤10% earlier observed. This may be explained by both the age structure of our population and the cutoff used to define hypertension. Over time the cutoff value for hypertension has been lowered from 160/95 mmHg to 140/90 mmHg. This inadvertently is bound to identify more people as having hypertension. Again, about 50% of our subjects were above sixty years of age and therefore could be classified as elderly people. As observed in this study and others from similar rural communities, there was an age-related increase in prevalence of hypertension [22]. The preponderance of elderly people in our study may be explained by the effect of rural-urban drift. Several of our rural communities have over the years witnessed rural-urban migration due to concentration of certain social amenities in such urban cities and/or in search of better jobs by the younger people thus leaving the elderly ones behind. Ike et al. [23] in a study among rural dwelling priests showed not only a higher prevalence of hypertension but also high level of unhealthy lifestyles and poor knowledge. 

Obesity was also a common risk factor in this study; however, there was a decrease in its prevalence with age. This could be due to high level of physical activity of the rural dweller arising from their farming activities and other household chores such as pounding and chopping of fire woods as well as poor socioeconomic status [24]. Despite the increasing prevalence trend, the awareness of many people about their cardiovascular risk status is still low [22, 25, 26]. The knowledge of the subjects about these risk factors and consequences were not ascertained in this study.

Dysglycaemia and elevated cholesterol were both relatively low in our subjects. Though we used a higher cutoff for total cholesterol, these findings are similar to the reports from the national survey on noncommunicable diseases in Nigeria carried out about two decades ago which gave a prevalence of 4.0% for elevated cholesterol (>5.2 mmol/L). Other studies carried out in Benin [27] and Port Harcourt [28] reported higher prevalence rates such as 27.6% and 35.9%, respectively, using cholesterol values >5.2 mmol/L. Application of cutoff level >5.2 mmol/L in this study would have given the prevalence of hypercholesterolaemia to be 14.8% (result not shown). This generally will show that while the prevalence is higher in urban dwellers, the burden may also be on the increase in rural dwellers. The reasons for higher burden among urban dwellers are quite understandable from their population dynamics. While the use of total cholesterol may poorly characterize and underestimate the burden of abnormalities of lipids, total cholesterol was used in this study because it has been shown that measurement of triglycerides showed no advantage over the use of cholesterol alone [29]. Hyperlipidaemia is becoming increasingly common as reported in some other African population (using cutoff level of >5.2 mmol/L) [9].

## 5. Conclusion

CVD risk factors are becoming increasing common in our rural communities. Of all the cardiovascular risk factors commonly assessed, hypertension and obesity are commoner in the rural community. The consequences of these risk factors in the face of low awareness call for greater public health awareness campaigns.

## 6. Limitations

The use of just two blood pressure readings taken at one sitting and the inability to do full lipid profile assessment may have affected the prevalence of hypertension and dyslipidaemia. The gender differences may also have been influenced by the gender skew towards female preponderance.

## Figures and Tables

**Table 1:** Characteristics of the population.

Variable	Overall (*n* = 858)	Men (*n* = 247)	Women (*n* = 611)	*P* value*
Age (yrs)	59.8 (9.9)	62.6 (9.2)	58.7 (9.9)	<.0001
Height (m)	1.56 (0.08)	1.62 (0.07)	1.54 (0.06)	<.0001
Weight (kg)	56.7 (13.2)	57.6 (11.3)	56.3 (13.9)	.177
Body mass index (Kg/m^2^)	23.2 (5.0)	21.9 (3.8)	23.7 (5.3)	<.0001
Waist circumference (cm)	84.6 (12.7)	83.1 (9.5)	85.3 (13.7)	.025
Systolic blood pressure (mmHg)	137.1 (25.7)	138.4 (24.1)	136.6 (26.3)	.347
Diastolic blood pressure (mmHg)	80.4 (15.6)	80.9 (15.7)	80.2 (15.5)	.532
Fasting blood glucose (mmol/L)	4.64 (1.7)	4.76 (2.2)	4.59 (1.4)	.262
Total cholesterol (mmol/L)^†^	4.59 (0.9)	4.41 (0.9)	4.65 (0.8)	.003

^†^(The sample sizes of the overall, men, and women groups for total cholesterol estimation were 593, 137, and 456 subjects, resp. The rest were returned as low values (i.e., <3.88 mmol/L) based on the measuring range of the machine). *P value between men and women.

**Table 2:** The prevalence of the risk factors by gender.

	Prevalence of cardiovascular risk factor (%)			
Cardiovascular risk factor	Men (*n* = 247)	Women (*n* = 611)	*χ* ^2^ (1)^†^	*P* value
Hypertension	124 (50.2)	274 (44.8)	1.821	.177
Generalized Obesity*	52 (21.1)	205 (33.6)	12.5	.0004
Abdominal Obesity	6 (2.4)	260 (42.6)	130.5	<.0001
Dysglycaemia	18 (7.3)	20 (3.3)	5.78	.016
Hypercholesterolaemia	6 (2.4)	26 (4.3)	1.165	.183

(*Comprises overweight and obese subjects. ^†^Yates correction applied).

**Table 3:** Cardiovascular risk factors by age group.

Age group (yrs)	*N*	Proportion (%) of individuals with abnormal risk factors within the age groups				
		Hypertension	Generalized obesity*	Abdominal obesity	Abnormal glucose tolerance	Hypercholesterolaemia
40–44	86	30 (34.9)	32 (37.2)	27 (31.4)	5 (5.8)	3 (3.5)
45–49	69	26 (37.7)	32 (46.4)	30 (43.5)	3 (4.3)	4 (5.8)
50–54	99	37 (37.4)	38 (38.4)	37 (37.4)	6 (6.1)	3 (3)
55–59	101	47 (46.5)	38 (37.6)	39 (38.6)	7 (6.9)	2 (2)
60–64	120	60 (50)	40 (33.3)	40 (33.3)	4 (3.3)	6 (5)
65–70	383	198 (51.7)	77 (20.1)	93 (24.3)	13 (3.4)	14 (3.7)

(*Comprises overweight and obese subjects).

## References

[1] Ahmad AM (1995). The health farm concept in the primary prevention of coronary artery disease. *Singapore Medical Journal*.

[2] Hennekens CH (1998). Increasing burden of cardiovascular disease: current knowledge and future directions for research on risk factors. *Circulation*.

[3] Kearney PM, Whelton M, Reynolds K, Muntner P, Whelton PK, He J (2005). Global burden of hypertension: analysis of worldwide data. *The Lancet*.

[4] Balarajan R (1991). Ethnic differences in mortality from ischaemic heart disease and cerebrovascular disease in England and Wales. *British Medical Journal*.

[5] Roderick PJ, Jones I, Raleigh VS, McGeown M, Mallick N (1994). Population need for renal replacement therapy in Thames regions: ethnic dimension. *British Medical Journal*.

[6] Raleigh VS (1997). Diabetes and hypertension in Britain’s ethnic minorities: implications for the future of renal services. *British Medical Journal*.

[7] Sobngwi E, Mbanya J-CN, Unwin NC (2002). Physical activity and its relationship with obesity, hypertension and diabetes in urban and rural Cameroon. *International Journal of Obesity*.

[8] Christensen DL, Eis J, Hansen AW (2008). Obesity and regional fat distribution in Kenyan populations: impact of ethnicity and urbanization. *Annals of Human Biology*.

[9] Njelekela M, Negishi H, Nara Y (2001). Cardiovascular risk factors in Tanzania: a revisit. *Acta Tropica*.

[10] Pobee JO (1993). Community-based high blood pressure programs in sub-Saharan Africa. *Ethnicity & Disease*.

[11] Aspray TJ, Mugusi F, Rashid S (2000). Rural and urban differences in diabetes prevalence in Tanzania: the role of obesity, physical inactivity and urban living. *Transactions of the Royal Society of Tropical Medicine and Hygiene*.

[12] Motala AA, Omar MAK, Pirie FJ (2003). Epidemiology of type 1 and type 2 diabetes in africa. *Journal of Cardiovascular Risk*.

[13] Lasky D, Becerra E, Boto W, Otim M, Ntambi J (2002). Obesity and gender differences in the risk of type 2 diabetes mellitus in Uganda. *Nutrition*.

[14] Schröder H, Marrugat J, Elosua R, Covas MI (2003). Relationship between body mass index, serum cholesterol, leisure-time physical activity, and diet in a Mediterranean Southern-Europe population. *British Journal of Nutrition*.

[15] Vorster HH (2002). The emergence of cardiovascular disease during urbanisation of Africans. *Public Health Nutrition*.

[16] Swai ABM, McLarty DG, Kitange HM (1993). Low prevalence of risk factors for coronary heart disease in rural Tanzania. *International Journal of Epidemiology*.

[17] O'Brien E, Asmar R, Beilin L (2003). European Society of Hypertension recommendations for conventional, ambulatory and home blood pressure measurement. *Journal of Hypertension*.

[18] Zanchetti A, Chalmers J, Arakawa K (1993). Summary of 1993 World Health Organisation-International Society of Hypertension guidelines for the management of mild hypertension. Subcommittee of WHO/ISH Mild Hypertension Liaison committee. *British Medical Journal*.

[19] Alberti KGMM, Zimmet PZ (1998). Definition, diagnosis and classification of diabetes mellitus and its complications. Part 1: diagnosis and classification of diabetes mellitus. Provisional report of a WHO consultation. *Diabetic Medicine*.

[20] WHO (2000). Obesity: preventing and managing the global epidemic. Report of a WHO consultation. *World Health Organization—Technical Report Series*.

[21] NCEP (2002). Third Report of the National Cholesterol Education Program (NCEP) Expert Panel on Detection, Evaluation, and Treatment of High Blood Cholesterol in Adults (Adult Treatment Panel III) final report. *Circulation*.

[22] Addo J, Amoah AGB, Kwadwo KA (2006). The changing patterns of hypertension in Ghana: a study of four rural communities in the Ga District. *Ethnicity and Disease*.

[23] Ike SO, Arodiwe EB, Onoka CA (2007). Profile of cardiovascular risk factors among priests in a Nigerian rural community. *Nigerian Medical Journal*.

[24] Fezeu L, Minkoulou E, Balkau B (2006). Association between socioeconomic status and adiposity in urban Cameroon. *International Journal of Epidemiology*.

[25] Ofoegbu EN (2002). Obesity unawareness in type 2 diabetic patients (letter). *Diabetes International*.

[26] Ulasi II, Ijoma CK, Onwubere BJC, Arodiwe E, Onodugo O, Okafor C (2011). High prevalence and low awareness of hypertension in a market population in Enugu, Nigeria. *International Journal of Hypertension*.

[27] Ukoli FAM, Bunker CH, Fabio A (1997). Sex differences in serum lipids pattern and their correlates in a Nigerian urban elderly population. *Nigerian Medical Journal*.

[28] Akpa MR, Agomouh DI, Alasia DD (2006). Lipid profile of healthy adult Nigerians in Port Harcourt, Nigeria. *Nigerian Journal of Medicine*.

[29] Avins AL, Neuhaus JM (2000). Do triglycerides provide meaningful information about heart disease risk?. *Archives of Internal Medicine*.

